# Application of ultrasound-guided coaxial needle biopsy combined with gelatin sponge plugging in pediatric liver biopsy

**DOI:** 10.3389/fped.2025.1477711

**Published:** 2025-05-20

**Authors:** Keyu Zeng, Boyang Yu, Zhe Wu, Jiehong Zhou, Qiang Lu

**Affiliations:** ^1^ Department of Medical Ultrasound, West China Hospital of Sichuan University, Chengdu, China; ^2^ School of Life Science and Technology, University of Electronic Science and Technology of China, Chengdu, China

**Keywords:** ultrasound guidance, coaxial technique, gelatin sponge particle, children, safety

## Abstract

**Purpose:**

The aim of this study was to assess the performance of utilizing the coaxial technique in conjunction with gelatin sponge slurry plugging for ultrasound-guided liver biopsy in children.

**Methods:**

We conducted a retrospective study of children undergoing ultrasound-guided coaxial liver biopsy at our institution between March 2020 and March 2025. Participants were stratified into two intervention groups: those receiving gelatin sponge tract embolization vs. batroxobin administered through needle tract. Through comprehensive electronic medical record review, we systematically extracted and compared the following outcome measures: (a) overall complication rates, (b) hemorrhage rates.

**Results:**

This study included 48 children, with 30 allocated to the gelatin sponge group and 18 to the batroxobin group. The gelatin sponge group demonstrated 9 complications (30.0%, 9/30), consisting of 5 pain events and 4 febrile episodes, while the batroxobin group experienced 6 complications (33.3%, 6/18), including 2 hemorrhage cases, 3 pain events, and 1 febrile episode. The overall complication rates showed no statistically significant difference between groups (30.0% vs. 33.3%, *P* = 0.809). However, a significant divergence was observed in hemorrhage incidence, with the gelatin sponge group demonstrating superior safety (0% vs. 11.1%, *P* = 0.044).

**Conclusion:**

Compared to batroxobin tract injection, coaxial technique in conjunction with gelatin sponge slurry plugging significantly reduced hemorrhagic complications in liver biopsies for children, demonstrating superior safety. The finding supported its adoption as the preferred hemostatic method in children undergoing percutaneous liver biopsy.

## 1 Introduction

Liver diseases encompass a diverse array of conditions, constituting some of the most prevalent and complex disorders encountered in contemporary clinical practice. Recent technological advancements have significantly enhanced the efficacy of imaging modalities in diagnosing the majority of liver diseases. Nonetheless, certain hepatic conditions may still necessitate pathological examination to acquire essential clinical insights for diagnosis, treatment, and prognosis, particularly in cases where imaging results are atypical or when specific therapeutic interventions are indicated (1). Although liver biopsy is generally considered as a minimally invasive procedure, it remains an invasive intervention associated with considerable risks (1, 2). Current literature has documented various post-biopsy complications, with hemorrhage being particularly significant (3).

The prevalence of hemorrhage following liver biopsy ranges from approximately 0.32%–1.9%, with increased rates observed in individuals presenting with abnormal coagulation profiles, liver cirrhosis, and in children (4, 5). The extant literature concerning the safety of percutaneous liver biopsy in children generally suggests that, despite variations in study cohorts and biopsy methodologies, the incidence of complications in children post-biopsy is higher compared to adults. Reported complication rates vary from 5%–28%, with hemorrhage occurring in 18% of cases (6). The adoption of the plugged liver biopsy (PLB) technique has demonstrated improvements in the safety of the biopsy procedure. PLB, which utilizes coaxial technology in combination with gelatin sponge occlusion, offers significant advantages in reducing bleeding-related complications following liver biopsy in patients predisposed to post-biopsy hemorrhage (7, 8).

However, there is a lack of studies on children undergoing liver biopsy, particularly concerning the use of gelatin sponge. Although prior investigations have primarily evaluated gelatin sponge pledgets, the efficacy of gelatin sponge particle slurry remains systematically unexamined in this vulnerable population. To address this clinical uncertainty, we conducted a retrospective study to assess the safety and technical feasibility of coaxial needle biopsy combined with gelatin sponge particle slurry for tract embolization following percutaneous liver biopsy in children.

## 2 Materials and methods

### 2.1 Patient selection

This retrospective study received approval from the Ethics Committee of West China Hospital of Sichuan University (Approval Number: 20241721). We retrospectively gathered data on patients who underwent ultrasound-guided liver biopsy at our institution between March 2020 and March 2025. Patients were excluded from the study if they met any of the following criteria: (a) age ≥18 years; (b) biopsy conducted without post-biopsy hemostatic measures; (c) anticoagulant therapy administered within 7 days prior to the biopsy; (d) radiotherapy or chemotherapy received within 30 days preceding the biopsy; (e) missing or insufficient follow-up records; and (f) lack of a definitive pathological diagnosis. The children were stratified into two groups based on the type of post-biopsy hemostatic measures employed: the gelatin sponge group (tract plugging with gelatin sponge particle slurry) and the control group (administration of batroxobin through the needle tract). Comprehensive clinical data were systematically collected, encompassing variables such as age, gender, indications for liver biopsy, usage of anticoagulant medications, pre-biopsy coagulation profiles, the number of biopsy punctures performed, the quantity of liver tissue cores obtained, and the administered dosage of gelatin sponge and batroxobin.

### 2.2 Procedure technique

Before conducting the biopsy procedure, it was essential to conduct a comprehensive review of the children's medical history to identify any contraindications for percutaneous liver biopsy. Additionally, it was imperative to furnish the guardian with detailed information concerning the procedure's objectives, methodology, and potential risks that may occur during and after the procedure. Obtaining informed consent from the guardian was mandatory, and their signature had to be secured.

**Figure 1:**
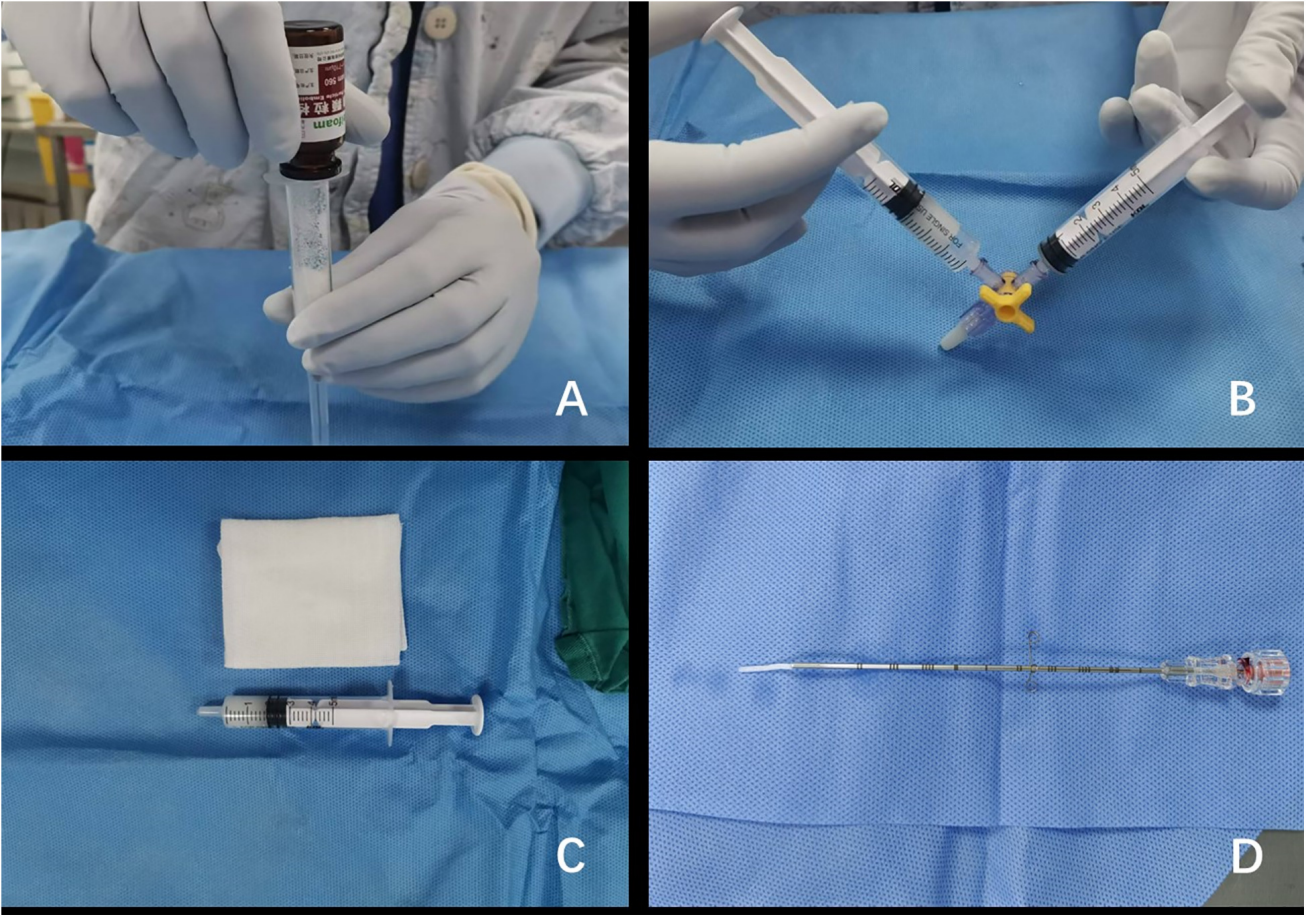
Preparation of gelatin sponge particle slurry. **(A)** The gelatin sponge particles were poured into a 5 ml syringe. **(B)** The gelatin sponge particles were uniformly mixed with 2 ml of normal saline using a three-way stopcock. **(C)** The gelatin sponge particle slurry was properly prepared and available for immediate intraoperative use. **(D)** A uniform strand of gelatin sponge particle slurry was precisely extruded from the coaxial needle assembly.

All percutaneous liver biopsies were performed under strict aseptic conditions by fellowship-trained interventional radiologists, each possessing >10 years of specialized experience. The procedures utilized standardized ultrasound guidance systems: either the IU-22 (Philips Healthcare, Eindhoven, Netherlands) with C5-1 sector probe or Resona 7 (Mindray Medical, Shenzhen, China) with SC6-1 sector probe, operating at 2–5 MHz frequencies. Anesthesia modality (general or local) was determined through multidisciplinary evaluation considering age of the child, cooperation level, and lesion characteristics. For gelatin sponge slurry preparation: (a) Medical-grade absorbable gelatin sponge particles (560–710 μm, Alicon, Hangzhou, Zhejiang, China) were aseptically poured into a 5 ml syringe; (b) Air evacuation was achieved through controlled plunger compression; (c) The Tessari mixing technique was employed to homogenize particles with 3 ml sterile saline; (d) Final slurry volume (2–3 ml) was verified and available for use (9) (Figure 1).

**Figure 2:**
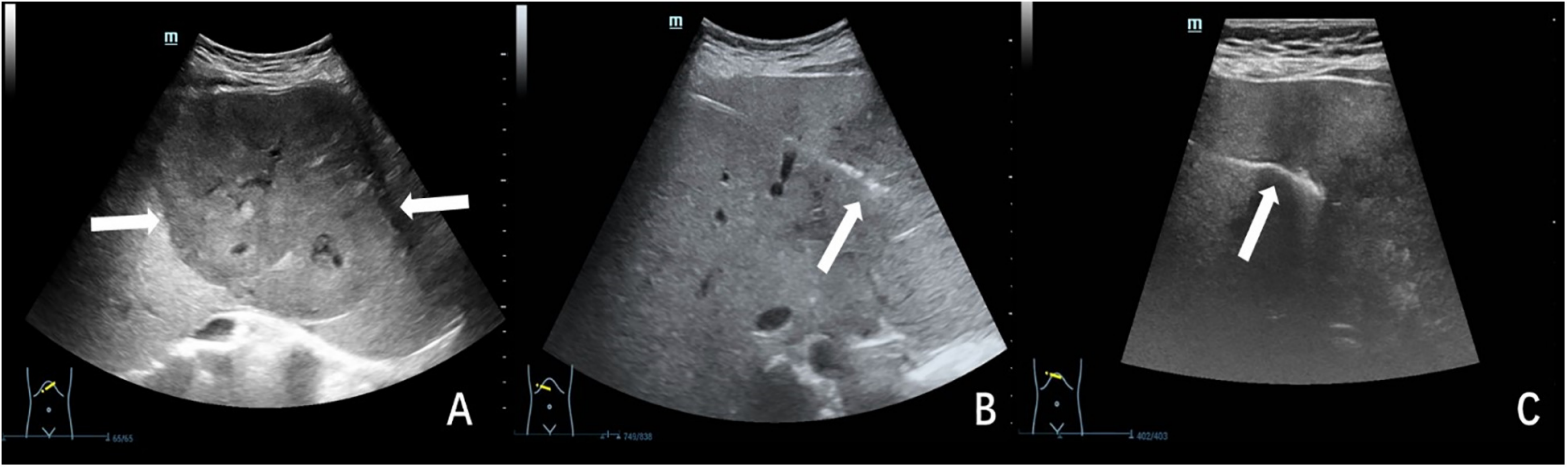
Ultrasound-guided plugged liver biopsy with gelatin sponge particle slurry embolization in a 17-year-old female child with indeterminate hepatic mass. The child's hemostatic profile was unremarkable, meeting all predetermined criteria for safe performance of percutaneous biopsy procedures. **(A)** Ultrasonographic examination revealed a large (11.3 × 8.6 cm), hypoechoic hepatic mass (indicated by arrow) located at the junction of the left and right hepatic lobes. **(B)** Under real-time ultrasonographic guidance, percutaneous biopsy of the hepatic mass was performed utilizing an 18-gauge cutting needle. The needle trajectory and tip position (indicated by arrow) were continuously visualized throughout the procedure. **(C)** The biopsy needle tract was meticulously plugged using gelatin sponge particle slurry. Ultrasound monitoring confirmed proper gelatin sponge deposition, visualized as a continuous hyperechoic linear structure (arrow) along the biopsy tract. The histopathologic analysis indicated a hepatocellular adenoma.

**Figure 3:**
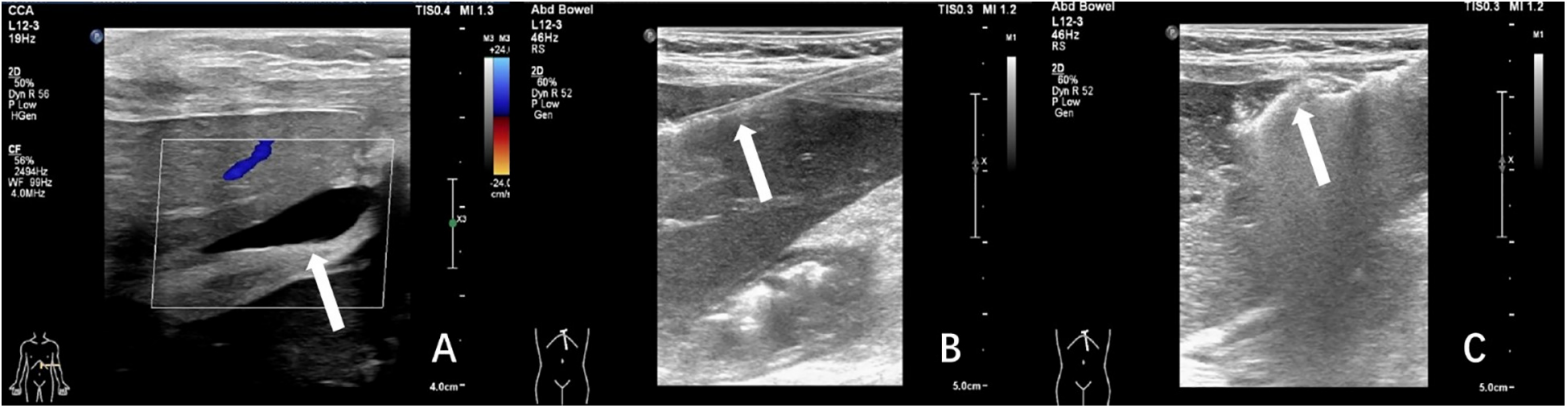
Ultrasound-guided plugged liver biopsy with gelatin sponge particle slurry embolization in a 3-year-old female child with post-transplant hepatic dysfunction. The child's laboratory results revealed elevated levels of total bilirubin (63.5 umol/L), alanine aminotransferase (592 IU/L), aspartate aminotransferase (1,445 IU/L). **(A)** Ultrasonography demonstrated anechoic fluid collection(arrow) localized in the surrounding region of the hepatic graft. **(B)** Under real-time ultrasonographic guidance, percutaneous core biopsy of the transplanted liver parenchyma was successfully performed utilizing an 18-gauge cutting needle (arrow denoted needle tip position). **(C)** The needle tract was plugged with gelatin sponge slurry which was shown as a hyper-echogenic line(arrow). The histopathologic analysis indicated drug-induced liver injury.

Preprocedural planning involved comprehensive abdominal ultrasonography to determine the optimal site for percutaneous access, supplemented by color Doppler mapping to identify and circumvent major vascular structures. Under continuous real-time ultrasound guidance, employing both B-mode and Doppler modalities, a 17-gauge coaxial introducer needle (Bard Peripheral Vascular Inc., Tempe, AZ, USA) was accurately advanced to the periphery of the predetermined target lesion. Upon removal of the stylet, tissue acquisition was conducted using an 18-gauge automated cutting needle (Bard Peripheral Vascular Inc., Tempe, AZ, USA), with specimens collected for histopathological analysis (Figures 2, 3 for representative imaging). For hemostatic management, two approaches were utilized: 1. Gelatin Sponge Group: (a) The prepared syringe containing gelatin sponge slurry was carefully aligned with the coaxial sheath; (b) The slurry was gradually injected under sonographic monitoring; (c) A blunt-tipped core needle was used to further facilitate the deposition of the gelatin sponge slurry at the biopsy site by pushing the stylet. 2. Batroxobin Group: (a) Following the biopsy procedure, 1 IU of batroxobin (Beijing Tobishi Pharmaceutical Co., Ltd., Beijing, China) was administered, reconstituted with 2 ml of normal saline, via the coaxial sheath; (b) Needle tract dwell time of 30 s before removal.

Immediately following the biopsy procedure, a comprehensive ultrasonographic evaluation was conducted to assess for acute complications, such as parenchymal bleeding or injury to adjacent organs. Standard postprocedural care included: (a) thorough disinfection of the biopsy site followed by the application of continuous manual pressure for 30 min to achieve hemostasis, and (b) a repeat ultrasound examination after the compression period to confirm the absence of delayed bleeding. Children were subsequently transferred to the ward for continuous cardiac monitoring while maintaining strict bed rest in a supine position for 24 h. Detailed discharge instructions emphasized: (a) avoidance of strenuous activity for 14 days, (b) recognition of warning signs, such as increasing abdominal pain, pallor, or lethargy, and (c) immediate medical consultation if any concerning symptoms developed. Guardians received both verbal and written guidance regarding these postoperative precautions prior to discharge.

### 2.3 Assessment criteria

The occurrences of post-biopsy complications were compared between the gelatin sponge group and the batroxobin group to assess the efficacy of utilizing the coaxial technique in conjunction with gelatin sponge particle slurry for tract plugging following liver biopsies in children.

In this study, post-biopsy complications were systematically defined as any adverse clinical or imaging findings occurring during the postprocedural recovery period that deviated from the expected healing course. Through retrospective review of comprehensive medical records, we documented all complications. Hemorrhage, a principal complication of percutaneous liver biopsy, was specifically defined by meeting ≥1 of the following objective criteria: (a) imaging confirmation of new perihepatic or intraperitoneal fluid collections (distinct from pre-existing ascites) on postprocedural ultrasound or CT; (b) laboratory evidence of acute blood loss (≥20% decline in hemoglobin or erythrocyte count)(3); (c) hemodynamic compromise manifesting as hypotension or persistent tachycardia (10); or (d) procedural bleeding requiring additional intervention (e.g., transarterial embolization) after failure of initial hemostatic measures (core-tract plugging or gelatin sponge embolization). Post-biopsy complications were categorized into minor and major according to the Society of Interventional Radiology's guidelines (11). Established risk factors for post-biopsy hemorrhage were identified from prior literature (6, 7, 12) and included: (a) platelet count < 50 × 10⁹/L; (b) international normalized ratio (INR) > 1.5; (c) prolonged prothrombin time (PT) (>3 s beyond upper limit of normal); and (d) clinically significant ascites.

Two critical parameters were employed to assess procedural efficacy: the technical success rate and the specimen adequacy rate. Technical success was strictly defined as the completion of all planned needle passes with retrieval of at least one histologically evaluable core specimen. Specimen adequacy was determined through pathological assessment, requiring sufficient tissue quantity and quality to enable definitive diagnosis. Cases where sampling limitations precluded conclusive pathological interpretation were classified as inadequate according to established histological criteria (13).

### 2.4 Statistical analysis

Statistical analyses were performed using SPSS software (version 25.0). Normally distributed continuous variables were expressed as mean ± standard deviation (SD) and compared using independent samples *t*-tests. Non-normally distributed continuous variables were presented as median (interquartile range, IQR) and analyzed using Mann–Whitney *U* tests. Categorical variables were reported as number (percentage) and compared using chi-square (*χ*²) tests. A two-tailed *P*-value < 0.05 was considered statistically significant.

## 3 Results

### 3.1 Patient characteristics

**Figure 4:**
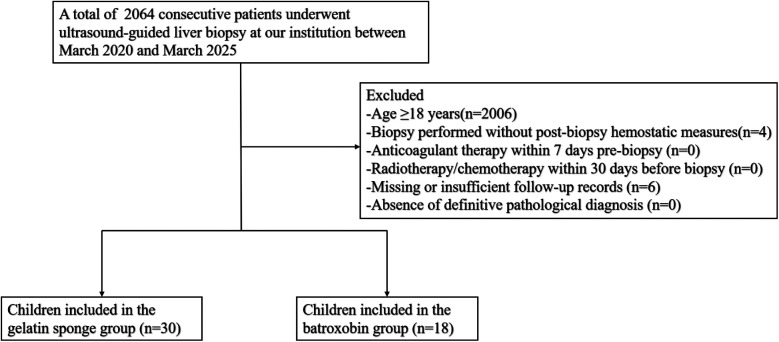
Flowchart showing inclusion of children undergoing percutaneous liver biopsy.

Between March 2020 and March 2025, a total of 48 children were included in this study, with 30 allocated to the gelatin sponge group and 18 to the batroxobin group. Figure 4 displays the inclusion flowchart for children. The gelatin sponge group of children comprised 14 males and 16 females, with a mean age of 8.1 ± 6.1 years. The batroxobin group of children comprised 15 males and 3 females, with a mean age of 11.2 ± 5.7 years. The mean preparation time for gelatin sponge granule slurry was 79.3 ± 18.6 s. The gelatin sponge group had a significantly lower mean age than batroxobin group (8.1 ± 6.1 vs. 11.2 ± 5.7 years, *P* = 0.025). Moreover, a statistically significant difference in sex distribution was observed between the two groups (*P* = 0.012). Detailed clinical characteristics of the children was presented in Table 1. All 48 children (100%) yielded diagnostically adequate specimens, with pathological diagnoses detailed in Table 2.

**Table 1:** Comparison of clinical characteristics of gelatin sponge group and batroxobin group in children.

Variable^a^	Gelatin sponge group	Batroxobin group	*P* value
Age (years)	8.1 ± 6.1	11.2 ± 5.7	0.025
Weight(kg)	39.1 ± 20.9	40.2 ± 18.6	0.932
Gender			
Male	14 (46.7)	15 (83.3)	0.012
Female	16 (53.3)	3 (16.7)	
Anesthesia modality			
General anesthesia	11 (36.7)	5 (27.8)	0.527
Local anesthesia	19 (63.3)	13 (72.2)	
Indications for biopsy			
Suspected liver malignancy	12 (40.0)	6 (33.3)	0.094
Abnormal liver function after liver transplantation	15 (50.0)	5 (27.8)	
Unexplained abnormal liver function	3 (10.0)	6 (33.3)	
Unexplained liver cirrhosis	0	1 (5.6)	
Biopsy target			
Focal liver lesion	12 (40.0)	6 (33.3)	0.644
Liver parenchyma	18 (60.0)	12 (66.7)	
Risk factors			
Platelet count < 50 × 10^9^/L	0	0	0.797
INR > 1.5	4 (13.3)	2 (11.1)	
Prolonged PT	4 (13.3)	4 (22.2)	
Ascites	5 (16.7)	3 (16.7)	
Number of punctures	2 (2,3)	2 (1,2)	0.096
Number of tissue cores	2 (2,3)	2 (1,2)	0.081
Technical success	30 (100)	18 (100)	>0.999
Adequate specimen	30(100)	18(100)	>0.999

INR, international normalized ratio; PT, prothrombin time.

^a^ Expressed as mean ± standard deviation, median (interquartile range) or number (percent, %).

**Table 2:** Pathological diagnoses of children in gelatin sponge group and batroxobin group.

Pathological diagnoses^a^	Gelatin sponge group	Batroxobin group
Hepatoblastoma	4 (13.3)	2 (11.1)
Hepatocellular adenoma	2 (6.7)	0
Tuberculosis	1 (3.3)	0
Burkitt's lymphoma	1 (3.3)	0
Angiosarcoma	1 (3.3)	0
Metastatic gastrointestinal stromal tumor	0	1 (5.6)
Hemangioendothelioma	1 (3.3)	0
Inflammatory pseudotumor	0	2 (11.1)
Nodular cirrhosis	1 (3.3)	3 (16.7)
Acute rejection	7 (23.3)	2 (11.1)
Chronic rejection	6 (20.0)	3 (16.7)
Drug-induced liver injury	3 (10.0)	1 (5.6)
Mild chronic hepatic inflammation	3 (10.0)	2 (11.1)
Metabolically associated fatty liver disease	0	2(11.1)

^a^ Expressed as number (percent, %).

### 3.2 Post-biopsy complications

The aggregate post-biopsy complication rate across the study cohort was 31.3% (15/48), with no statistically significant difference observed between the gelatin sponge and batroxobin groups [30.0% (9/30) vs. 33.3% (6/18), *P* = 0.809]. However, a clinically significant disparity emerged in hemorrhagic complications, with the batroxobin group demonstrating substantially higher incidence rates [11.1% (2/18) vs. 0% (0/30); *P* = 0.044], as detailed in Table 3.

**Table 3:** Complications between the gelatin sponge group and batroxobin group.

Complications^a^	Gelatin sponge group	Batroxobin group	*P* value
Hemorrhage	0	2 (11.1)	0.044
Pain	5 (16.7)	3 (16.7)	>0.999
Febrile episode	4 (13.3)	1 (5.6)	0.091

^a^ Expressed as number (percent, %).

Within the gelatin sponge group, five children (16.7%) experienced procedure-related pain, of which two cases resolved spontaneously while three required analgesic intervention. Additionally, four febrile episodes (13.3%) were documented, with peak temperatures reaching 39°C. All febrile cases were effectively managed through conservative measures, including physical cooling techniques and oral antipyretic administration, with complete resolution within 24 h post-procedure.

**Figure 5:**
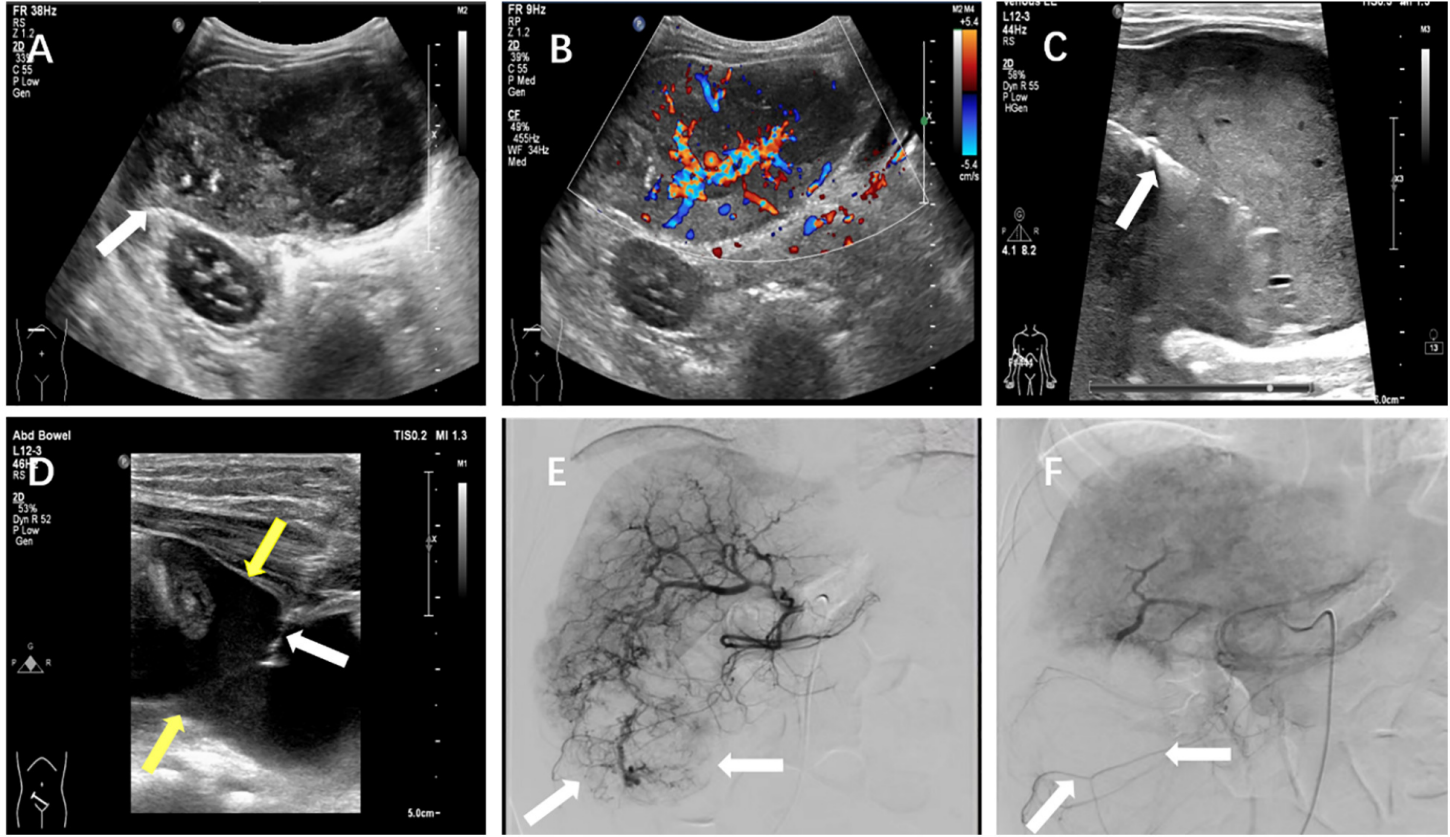
A severe postprocedural hemorrhage following liver biopsy in a 3-year-old child with hepatoblastoma. **(A)** Grayscale ultrasound demonstrated a heterogeneous hypoechoic mass (arrow) measuring approximately 8.1 × 4.5 cm in the hepatic parenchyma. **(B)** Color Doppler imaging revealed marked intralesional vascularity. **(C)** Ultrasound-guided core needle biopsy of the hepatic mass was performed under real-time visualization (arrow indicated biopsy needle trajectory). **(D)** On postoperative day 4, emergent ultrasound showed significant hemoperitoneum, with diagnostic paracentesis yielding non-coagulating blood (white arrow: paracentesis needle; yellow arrow: intraperitoneal hemorrhage). **(E)** Day 5 angiographic evaluation identified a hyper-vascular mass (arrow) in the right hepatic lobe. **(F)** Post-embolization angiography confirmed successful devascularization of the lesion (arrow indicated residual tumor blush reduction).

The batroxobin group demonstrated three cases (16.7%) of self-limiting pain requiring no medical intervention, one febrile episode (5.6%) managed effectively with physical cooling and oral antipyretics, and two significant hemorrhagic complications (11.1%). The first hemorrhage occurred in a 3-year-old female liver transplant recipient with pre-existing hepatic insufficiency, where 30 min post-procedural ultrasonography identified a perihepatic fluid collection. This was successfully treated with intravenous hemostatic agents and packed red blood cell transfusion. The second, more severe hemorrhagic event developed in a 3-year-old child (male) undergoing biopsy of a right hepatic lobe mass (later histologically confirmed as hepatoblastoma). On postoperative day 4, the child manifested acute syndrome characterized by severe pain, pallor and cold extremities, and an acute hemoglobin decline to 68 g/L. Emergency abdominal ultrasound revealed substantial hemoperitoneum, with diagnostic paracentesis obtaining non-coagulating blood. Subsequent angiography on day 5 identified a hypervascular mass in the right hepatic lobe, which was successfully embolized using gelatin sponge. Combined with blood transfusion, fluid resuscitation, and peritoneal drainage, this comprehensive approach resulted in full recovery. Details for this severe hemorrhage were illustrated in Figure 5.

## 4 Discussion

This investigation aimed to assess the clinical efficacy and safety profile of ultrasound-guided coaxial needle biopsy combined with gelatin sponge particle slurry embolization in children. In contrast to conventional gelatin sponge pledget techniques documented in prior studies (6, 12, 14), our results demonstrated that gelatin sponge particle slurry represented a technically viable and potentially superior alternative for achieving effective transhepatic tract occlusion in children.

The principal clinical value of gelatin sponge particle slurry embolization resides in its demonstrated capacity to prevent post-biopsy hemorrhagic complications, as evidenced by our findings of significantly lower hemorrhage rates compared to batroxobin administration (0% vs. 11.1%, *P* = 0.044). This outcome substantiated the superior hemostatic reliability achieved through coaxial deployment of gelatin sponge particle slurry. While the observed difference in febrile episodes between groups did not attain statistical significance (13.3% vs. 5.6%, *P* = 0.091), the numerically higher incidence in the gelatin sponge group may be attributed to two biologically plausible mechanisms: (a) xenogeneic immune stimulation from porcine-derived collagen components, known to induce proinflammatory cytokine release and complement cascade activation (15); and (b) localized ischemic effects from mechanical embolization, potentially causing parenchymal necrosis and absorption fever (15, 16). Our study documented a clinically significant hemorrhagic event in a 3-year-old child with hepatoblastoma (tumor dimensions: 8.1 × 4.5 cm) exhibiting multiple high-risk characteristics: (a) hypervascular tumor architecture, (b) substantial lesion volume, and (c) compromised postoperative immobilization (17). The child's low body weight (14 kg) markedly amplified the hemodynamic consequences of bleeding, underscoring the particular vulnerability of children. This case was successfully managed through emergent transarterial embolization and targeted blood product replacement, yielding complete recovery. Based on these observations, we recommended enhanced preventive protocols for young, low-weight children undergoing liver biopsy: (a) rigorous preoperative evaluation of biopsy indications, (b) intraoperative hemostatic measures including extended manual compression and coaxial tract embolization, and (c) postoperative bed rest extension to mitigate major hemorrhagic complications.

Percutaneous liver biopsy demonstrates markedly different risk profiles between children and adult populations, with existing literature reporting substantially higher complication rates (5%–28% vs. ∼1%) and lower diagnostic accuracy (<86% vs. 98%) in children compared to adults (6). This disparity is particularly pronounced in low-weight children, as evidenced by a study documenting a 10% complication rate including one mortality in a 2.6 kg infant, with strong inverse correlation between weight and procedural safety (18). These findings underscore the critical need for optimized techniques in children. The evolution of hemostatic methods has significantly improved safety of biopsy for children (19). Multiple studies have validated the efficacy of gelatin sponge tract occlusion, with Lungren et al. reporting zero hemorrhagic complications among 67 high-risk children (<10 kg) with coagulopathies or thrombocytopenia (6). Comparative research between transjugular liver biopsy (TJLB) and PLB demonstrated comparable safety profiles (2.6% vs. 3.3% complications, respectively), though PLB avoids radiation exposure (12). Previous studies using gelatin sponge pledgets have shown the effectiveness of gelatin sponges in decreasing complications post liver biopsy for children. However, traditional gelatin sponge pledget techniques present practical limitations, requiring complex preparation and additional personnel that may delay emergency interventions. Our study introduced a technical refinement (gelatin sponge particle slurry) that addressed these limitations while maintaining hemostatic efficacy. This innovation offered key advantages: the preparation process was straightforward and expeditious, taking only an average of one minute. In instances of acute hemorrhage, it can be promptly prepared for immediate administration (20).

Gelatin sponge particles demonstrate distinct physicochemical properties characterized by an interconnected porous microstructure and pronounced hydrophilic characteristics, endowing them with exceptional fluid absorption capacity. When applied to hemorrhagic sites, these particles rapidly absorb blood components through capillary action, leading to: (a) local hemodynamic alterations that reduce active bleeding, and (b) mechanical expansion that produce both spatial occlusion of the needle tract and direct compressive forces on adjacent vasculature. This dual mechanism which combining passive absorption with active physical compression facilitates reliable hemostasis through: (a) platelet aggregation and activation at the particle surface, (b) formation of a stable physical barrier, and (c) maintenance of sustained mechanical pressure until natural coagulation pathways are established (21). In addition, the gelatin sponge slurry demonstrates optimal characteristics as a fluid embolic agent, combining excellent tissue conformability with facile administration through coaxial needle systems. Its rheological properties enable precise adaptation to irregular wound geometries, making it particularly suitable for percutaneous hemostatic applications. When augmented with thrombin, the composite materials exert dual hemostatic mechanisms: (a) mechanical occlusion through particle expansion and (b) biochemical activation of coagulation cascades (19). Despite these advantages, several safety considerations warrant discussion. As a xenogeneic material, gelatin sponge carries potential risks including hypersensitivity reactions, microvascular occlusion leading to focal necrosis, and secondary infection/abscess formation (22–24). Theoretical concerns regarding venous migration and thromboembolism remain controversial. While particulate entry into hepatic or portal circulation could potentially predispose to pulmonary embolism, no confirmed cases have been documented in the literature (25–27). Clinical differentiation between procedure-related vascular injury and sponge-induced thrombosis proves challenging due to overlapping radiographic features (25). To date, no published cases of pulmonary embolism directly attributable to gelatin sponge embolization have been reported. The following preventive strategies are recommended to mitigate this theoretical risk: (a) meticulous needle trajectory planning to avoid venous structures; (b) confirmation of needle tip position prior to injection; (c) slow, controlled administration of gelatin sponge slurry; (d) selection of larger particle sizes; (e) real-time monitoring by mixing gelatin sponge with contrast agent, allowing visualization of flow direction during injection (22). Moreover, a significant correlation is identified between the incidence of complications and the size of gelatin sponge particles, with larger particles associated with a reduced risk. Additionally, gelatin sponge is capable of complete degradation within the human body, rendering it a safe and reliable option (15, 28). While demonstrating superior efficacy to conventional methods, the cost-effectiveness profile of gelatin sponge slurry requires consideration, particularly in resource-limited settings. Future development of standardized, cost-optimized formulations may enhance accessibility without compromising safety or performance.

This study was subject to certain limitations. First, the sample size was small and sourced from a single center, necessitating further validation of the research findings in a larger population to ensure reproducibility and generalizability. Second, the retrospective design of the study posed challenges in standardizing long-term follow-up assessments, with clinical information on children only being evaluated when accessible.

## 5 Conclusion

The application of ultrasound-guided coaxial technique combined with gelatin sponge slurry offers a promising strategy to efficiently reduce the occurrence of bleeding complications following liver biopsies in children, providing a simple procedure while ensuring utmost safety. Consequently, its clinical implementation holds substantial potential for widespread acceptance.

## Data Availability

The data analyzed in this study is subject to the following licenses/restrictions: Data set from West China Hospital of Sichuan University. Requests to access these datasets should be directed to ken18281180612@163.com.
